# Editorial: Integrative approaches with BCI and robotics for improved human interaction

**DOI:** 10.3389/frobt.2026.1785247

**Published:** 2026-02-06

**Authors:** Hammad Nazeer, Farzan M. Noori, Rayyan Azam Khan

**Affiliations:** 1 Department of Mechatronics Engineering, Air University, Islamabad, Pakistan; 2 Department of Informatics and RITMO, University of Oslo, Oslo, Norway; 3 Paul Albrechtsen Research Institute, CancerCare Manitoba, Winnipeg, MB, Canada; 4 Department of Electrical and Computer Engineering, University of Manitoba, Winnipeg, MB, Canada

**Keywords:** artificial intelligence, brain-computer interface BCI, deep learning, hybrid-BCI, machine learning, rehabilitation, robotics

With the advancement in neurotechnology the human-machine interaction has been redefined, specifically with progress in brain-computer interface (BCI) systems (Zhang et al., 2023). By translating neural activity directly into machine-executable commands, BCIs are unlocking unprecedented possibilities in healthcare, assistive robotics, brain-controlled therapy and neurorehabilitation (Karikari and Koshechkin, 2023; Nazeer et al., 2025). Among all BCI types, invasive BCIs have better signal quality and precision, and non-invasive BCIs are cheaper, comfortable, and suitable for consumer and clinical use (Naseer and Hong, 2015). In non-invasive BCIs, intensive research has been performed, still there is need to cope with lower resolution and noise which can be improved with pre-processing, artificial intelligence (AI) for robotic applications (Hong et al., 2018; Hanafi et al., 2023).

This Research Topic explores the synergy between non-invasive neuroimaging modalities like EEG and fNIRS, and robotics to nurture more natural and intuitive collaborations between humans and artificial intelligence. It is an effort to collect current contributions on improved applications of BCIs in mental workload monitoring, bridging neural signals and natural language, explainable artificial intelligence, and robotics application for motor rehabilitation.

This research collection is the result of contributions from 12 authors, 14 reviewers and editors from 11 countries (Pakistan, Canada, Norway, Singapore, Germany, China, Romania, Italy, Australia, United Kingdom and United States) from Medical Institutions, Academic Institutions and Research Centers.

The Research Topic mainly contributed in open access datasets, decoding neural signal precisely, exploring the dominant features in deep learning models, enhancing the ability of BCIs to operate effectively in diverse real-world environments and integrating explainable-AI with neural signals to perform contrastive learning. Here is the brief summary of main contributions which demonstrated the innovative approaches to solve current challenges, technical hurdles and enhance the diverse application of BCIs.

Data acquisition is a vital component for efficient and robust BCI-based application. This step results in robust datasets to develop BCIs for motor rehabilitation, such as controlling assistive and therapeutic robotic devices (Sun et al., 2025). Khan et al. contributed a structured and preprocessed functional near infrared spectroscopy (fNIRS) based open access dataset focused on lower limb motor imagery tasks involving the ankle knee joint. This is an effort to accentuate assistive and rehabilitation robotics and advanced machine learning models to decode movement intent from fNIRS signals. This also provides a way forward for transparent understanding and reproducible studies.

One of the challenges and emerging area of research in BCI based neurotechnology is task-specific performance of deep learning models with explainable. Camaret Ndir et al. proposed EEG-CLIP a contrastive learning framework that aligns EEG time-series data with corresponding clinical medical reports. This framework implemented few-shot and zero-shot decoding using textual prompts, allowing models to classify neural patterns using textual prompts without requiring task-specific training. This multimodal integration of neural data and text paves the way for more generalizable EEG representations, which results in analysis of diverse EEG decoding or training task-specific models with fewer samples.

In environments demanding rapid decision under pressure with extreme precision like aviation, cognitive state of pilot is a critical factor in safety (Albuquerque et al., 2020). Haseeb et al. demonstrated a passive BCI (pBCI) system designed to monitor pilots’ mental workload during real flight conditions. The system employed multinomial logistic regression with a ridge estimator to achieve 84.6% mean accuracy in detecting workload levels using a dry-electrode EEG system. This work shows the potential for real-time BCI applications to mitigate human error in complex and dynamic scenarios.

In recent times, deep learning models become more popular in EEG decoding but understanding what these models learn and which features are more dominant in learning are important for trust and application diversity. Schirrmeister and Ball proposed novel EEG-InvNet and EEG-CosNet interpretability methods to explore features learned by the complete network. These methods allow researchers to visualize neural signals and identify expected and unexpected features, such as sub-delta frequency patterns, which may classify pathological and nonpathological EEG. The study is a step forward in embedding explainable AI in neurotechnology which may have the potential of visualization to understand the network prediction function without relying on specific predefined features.

These contributions explored and identified the directions of future research in integrations of BCI, robotics and human interaction. The Research Topic demonstrate that human interaction or BCI may step-forward with the integration of single and multi-modal systems with advanced robotics and artificial intelligence. By addressing these challenges in processing pipelines, learning model interpretability, data accessibility, explainable AI and diverse applications, we may move closer to establish state-of-the-art neurotechnology. This may help in improving the quality of life for users across various sectors and daily life by intuitively responding to our cognitive and physical needs.

## References

[B1] Albuquerque I. Tiwari A. Parent M. Cassani R. Gagnon J.-F. Lafond D. (2020). WAUC: a multi-modal database for mental workload assessment under physical activity. Front. Neurosci. 14 (December), 549524. 10.3389/fnins.2020.549524 33335465 PMC7736238

[B2] Hanafi S. A. Bin Abdul Rahman H. Pertiwi D. A. A. Muslim M. A. (2023). Brain computer interface (BCI) machine learning process: a review. J. Electron. Technol. Explor. 1 (1), 29–35. 10.52465/joetex.v1i1.189

[B3] Hong K. S. Khan M. J. Hong M. J. (2018). Feature extraction and classification methods for hybrid FNIRS-EEG brain-computer interfaces. Front. Hum. Neurosci. 12 (June), 1–25. 10.3389/fnhum.2018.00246 30002623 PMC6032997

[B4] Karikari E. Koshechkin K. A. (2023). Review on brain-computer interface technologies in healthcare. Biophys. Rev. 15 (5), 1351–1358. 10.1007/s12551-023-01138-6 37974976 PMC10643750

[B5] Naseer N. Hong K.-S. S. (2015). FNIRS-based brain-computer interfaces: a review. Front. Hum. Neurosci. 9 (January), 1–15. 10.3389/fnhum.2015.00003 25674060 PMC4309034

[B6] Nazeer H. Naseer N. Khan M. J. Hong K.-S. (2025). “Noninvasive brain–computer interfaces using FNIRS, EEG, and hybrid EEG-FNIRS,” in Brain-Computer Interfaces (Elsevier), 297–326. 10.1016/B978-0-323-95439-6.00003-X

[B7] Sun Y. Chen X. Liu B. Liang L. Wang Y. Gao S. (2025). Signal acquisition of brain–computer interfaces: a medical-engineering crossover perspective review. Fundam. Res. 5 (1), 3–16. 10.1016/j.fmre.2024.04.011 40166113 PMC11955058

[B8] Zhang J. Li J. Huang Z. Huang D. Yu H. Li Z. (2023). Recent progress in wearable brain–computer interface (BCI) devices based on electroencephalogram (EEG) for medical applications: a review. Health Data Sci. 3 (January), 0096. 10.34133/hds.0096 38487198 PMC10880169

